# Automatic speech recognition for Telugu: a comparative analysis of Wav2Vec 2.0 model variants and hyperparameter tuning

**DOI:** 10.3389/frai.2026.1813668

**Published:** 2026-06-24

**Authors:** Anvita Manne, Nikhita James, Ishaan Jain, Jagalingam Pushparaj

**Affiliations:** Department of Quantum AI, School of Computer Science and Engineering, Vellore Institute of Technology, Vellore, India

**Keywords:** automatic speech recognition, fine-tuning, hyperparameters, low-resource languages, Telugu, Wav2Vec 2.0

## Abstract

Automatic speech recognition (ASR) systems for the Telugu language can significantly enhance speech-based interaction and digital accessibility. Although Telugu is among the top languages in the Dravidian family in terms of the number of speakers, there is a notable absence of standardized, noise-robust resources for Telugu ASR development. The existing systems are not specifically designed for low-resource languages like Telugu. The datasets available for Telugu are restricted to controlled environments and do not include noise interferences. Most existing ASR models are optimized for high-resource languages such as English, leading to poor generalization when applied to Telugu. In this work, we fine-tuned three pre-trained variants of the Wav2Vec 2.0 model: XLS-R 300M, XLSR-53, and XLS-R-1B on a curated 48.4-h Telugu speech corpus compiled from Mozilla Common Voice, OpenSLR, and the Hugging Face dataset. This study utilized a comprehensive ablation study on four hyperparameters to evaluate their recognition performance using word error rate (WER) and character error rate (CER) as evaluation metrics. Among the three models, the fine-tuned XLS-R-1B performed the best, achieving a WER of 36.23% and CER of 15.44%, followed by the XLSR-53 (WER 37.78%, CER 15.81%) and XLS-R-300M (WER 37.87%, CER 15.81%). An important consideration of the research was finding the right trade-off between a model's complexity and the accuracy of its performance. The results showed that the size of the model is important, and hyperparameter selection significantly influences the development of an effective ASR model in low-resource languages such as Telugu.

## 1 Introduction

India's linguistic landscape is unique, characterized by more than a hundred official languages. ASR serves as a fundamental technology in areas such as education, language preservation, and digital accessibility (Ghosh et al., 2020). Although substantial progress has been made in high-resource languages like English (Shi et al., 2021; Dar and Pushparaj, 2024; Song, 2019), low-resource languages such as Telugu remain underrepresented in the field due to the scarcity of high-quality labeled data and the complexity of the language's phonetic structure (Khan and Reddy, 2022). A significant challenge for Telugu ASR development is the lack of a large annotated speech corpus, a common challenge across many low-resource Indian languages (Mirishkar et al., 2023). Furthermore, the performance of the models is adversely affected by variations in speaking style, intonation, pronunciation, accent, and the speaker's dialect (Ahmad Dar and Pushparaj, 2025).

Recent work on Telugu dialect speech datasets and recognition using deep learning (DL) techniques highlights the importance of capturing dialectal diversity for improved ASR performance (Podila et al., 2025). Accordingly, there are four major dialect areas spread over this geographical area. The northern dialects are spoken in Telangana; the southern dialects are spoken in Rayalaseema and the two southernmost districts of Coastal Andhra; the eastern dialects are spoken in the three northern districts of Coastal Andhra. Standard Telugu is spoken in Central Andhra–particularly in the Guntur, Krishna, East Godavari, and West Godavari districts. Apart from the regional variations, Telugu contains a diverse set of phonemes, which include 16 vowels and 36 consonants. It includes both short and long versions of eight basic vowel sounds. Telugu consonants include plain stops, nasals, and approximants, as well as aspirated and breathy-voiced stops and retroflex consonants. These characteristics make Telugu phonetically rich and distinct, which in turn presents unique challenges for ASR systems, particularly in the context of speaker variability and dialectal diversity (Shivaprasad and Sadanandam, 2020).

The contributions of the presented work are

- Curated a 48.4-h multisource Telugu speech corpus that integrates and preprocesses three major data sources: Mozilla Common Voice, OpenSLR, and the Hugging Face dataset.
- Fine-tuned three variants of Wav2Vec 2.0 (XLS-R-300M, XLSR-53, and XLS-R-1B) pre-trained models on the curated Telugu corpus.
- Conducted a comprehensive ablation study for four major hyperparameters relevant to ASR performance (learning rate, hidden layer dropout, optimizer, and number of training epochs). Hyperparameters of interest were tested with a range of values.
- Evaluated the final fine-tuned models on an unseen Indic TTS (text-to-speech) dataset. This demonstrated the generalization capacity of the models to new speakers, new accents, and new contexts outside the training distribution.
- Evaluated the performance of the final fine-tuned models on a seen training dataset with added artificial noise.

The remainder of this paper is structured as follows: Section 2 provides a review of related work. Section 3 describes the methodology, including data preparation, model architecture, and evaluation metrics. Section 4 presents the experimental results and discusses the observations from the model evaluations. Section 5 presents a qualitative analysis of the model observation. Finally, Section 6 gives the conclusion of the study and suggests directions for future research.

## 2 Related studies

Speech analysis has been a major topic of study as a result of the tremendous transformation of several academic disciplines brought about by advances in DL. Applications including speech translation, ASR speech synthesis, ambient audio signal detection, and sound quality enhancement are all being actively investigated in current research. Various techniques have been used to improve ASR systems. Data augmentation is one of the most commonly used solutions, which involves introducing more variations to the data directly, making it more robust. The authors (Majhi and Saha, 2024) incorporated an attention network named BiSeq + AtM on the baseline bidirectional long short-term memory (BLSTM) networks and the Seq2Seq framework, using Mel-frequency cepstral coefficients (MFCCs) for feature extraction in an Odia ASR system. The authors Dar and Pushparaj (2025) employed multiple acoustic features of the speech signal to develop a recognition system for the low-resource Kashmiri language using a BLSTM network, achieving an accuracy of 85.25%. Chimalamarri and Sitaram (2021) proposed a system that treats each word as a sequence of linguistically derived subwords and includes parts of speech tags to provide additional grammatical context.

There is a growing need for ASR technologies in diverse low-resource languages like Telugu for bilingual communication. Gabor filters for audio-visual features and a multi-layer perceptron framework were used to intelligently adjust the weights of a six-dimensional feature vector. The Gabor filters and multi-state Hidden Markov Models (HMMs) provided a 5.5% increase in the average accurate rate of speech recognition. The traditional methods should be replaced by Convolutional Neural Networks (CNNs) and end-to-end models using BLSTM networks to handle complex data for audio-visual speech recognition (Saudi et al., 2019). In real-time, the process of audio recognition can be challenging when there is noise involved. Developing a robust Telugu ASR system that's resilient to noise variations should be the main focus. Khondkar et al. (2024) developed a public dataset called Voice Pre-processing and Quality Assessment Dataset to improve voice recognition by including real-time noisy environment conditions and made it available to use for advanced research. For Telugu speech recognition, MFCCs were used to extract the key features from Telugu speech, and the HMM was trained to recognize the different phonemes and words of the Telugu language accurately. Wang et al. (2020) developed an HMM-based accurate speech recognition system for English learning using MFCC for feature extraction to improve overall accuracy and response time. The K-Means clustering algorithm was used for vector quantization to generate the HMM template, and 2,000 English words were collected from 20 individuals. MATLAB was used for signal processing and evaluation, which concluded that the response time was 0.13 s and accuracy was above 90% even in noisy environments.

Speech enhancement involves improving speech quality by reducing background noise without degrading any information, which in turn helps in improved speech recognition. Balasubrahmanyam and Valarmathi (2024) explored the process of speech enhancement by adapting DL models to obtain more contextual feature information and improved results. This process showed a drop in root mean square error (RMSE) and peak signal-to-noise ratio values upon evaluation. Prabhavalkar et al. (2017) aimed to survey various end-to-end ASR models and contrast them with classic HMM-based systems. The models focus on combining all component models into a single network, making it easier to train, deploy, and maintain. They incorporate data augmentation and advanced architectures to achieve state-of-the-art performance but struggle with low-resource data and require an additional language model to improve robustness. On comparing, end-to-end models outperformed traditional hybrid HMM models with lower WER rates (Prabhavalkar et al., 2017).

Different DL techniques improve ASR robustness against domain mismatch. Each offers distinct advantages for specific applications. However, challenges include computational demands and vulnerability to sparse input data. Deep transfer learning addresses data scarcity and domain mismatch challenges in ASR but faces issues such as distribution shift, catastrophic forgetting, and hyperparameter optimization (Kheddar et al., 2024). The authors explored methods to improve accuracy in noisy environments, handle diverse accents and languages, and ensure contextual awareness to enhance the robustness and utilization of Speech-to-Text (STT) and TTS systems while addressing ethical concerns. Their findings show that DL methods such as CNNs, Recurrent Neural Networks (RNNs), and transformers outperform traditional methods in accuracy and contextual translation (Reddy et al., 2023). The inclusion of vision-language pretraining models has shown improvements in STT accuracy, particularly in the medical field (Huh et al., 2023). Their solution proposed a multimodal medical speech module that utilizes a vision-language pretrained model to correct errors and improve findings in general STT tasks using medical images. This model helped reduce WER and CER rates, demonstrating more accurate results. The authors Alsharhan and Ramsay (2023) used an HMM toolkit with the help of deep neural network (DNN) modules for Arabic speech recognition. They found that Egyptian Arabic was the easiest to recognize. Gulf and Arabic were frequently confused. Maghrebi speakers were often mistaken for Levantine. Another study Miah et al. (2024) explored the feasibility of sentiment analysis for foreign languages when converted to a base language first. However, problems include a lack of accuracy in identifying slang and correctly translating idioms or cultural nuances. Shadiev et al. (2020) explored a speech-enabled language translation model by integrating computer-aided translation technology with STT for enhancing comprehension, especially for those with low English proficiency in foreign lectures. These outcomes suggest that adding translation to STT technology can enhance students' learning outcomes.

Most existing ASR models are trained on large-scale corpora from high-resource languages like English, which makes them suitable for wide application in tools like voice assistants, transcription, and accessibility features. However, Telugu lacks large, diverse, and representative datasets, especially those that include real-world audio disturbances such as background noise, informal speech, and speaker variation (Kumar et al., 2025). Due to this limitation, we constructed a combined dataset from open-source Telugu corpora to enable the training of ASR models. This combined dataset was used to systematically train the models and evaluate their performance on Telugu speech recognition. We evaluate XLS-R-300M, XLSR-53, and XLS-R-1B architectures to assess their performance for Telugu speech recognition. During fine-tuning, an ablation study was conducted to examine the impact of four hyperparameters–learning rate, hidden dropout, optimizer, and epochs–each tested across three different values for every model. All models were evaluated using both WER and CER metrics to account for linguistic and phonetic accuracy.

## 3 Methodology

**Figure 1:**
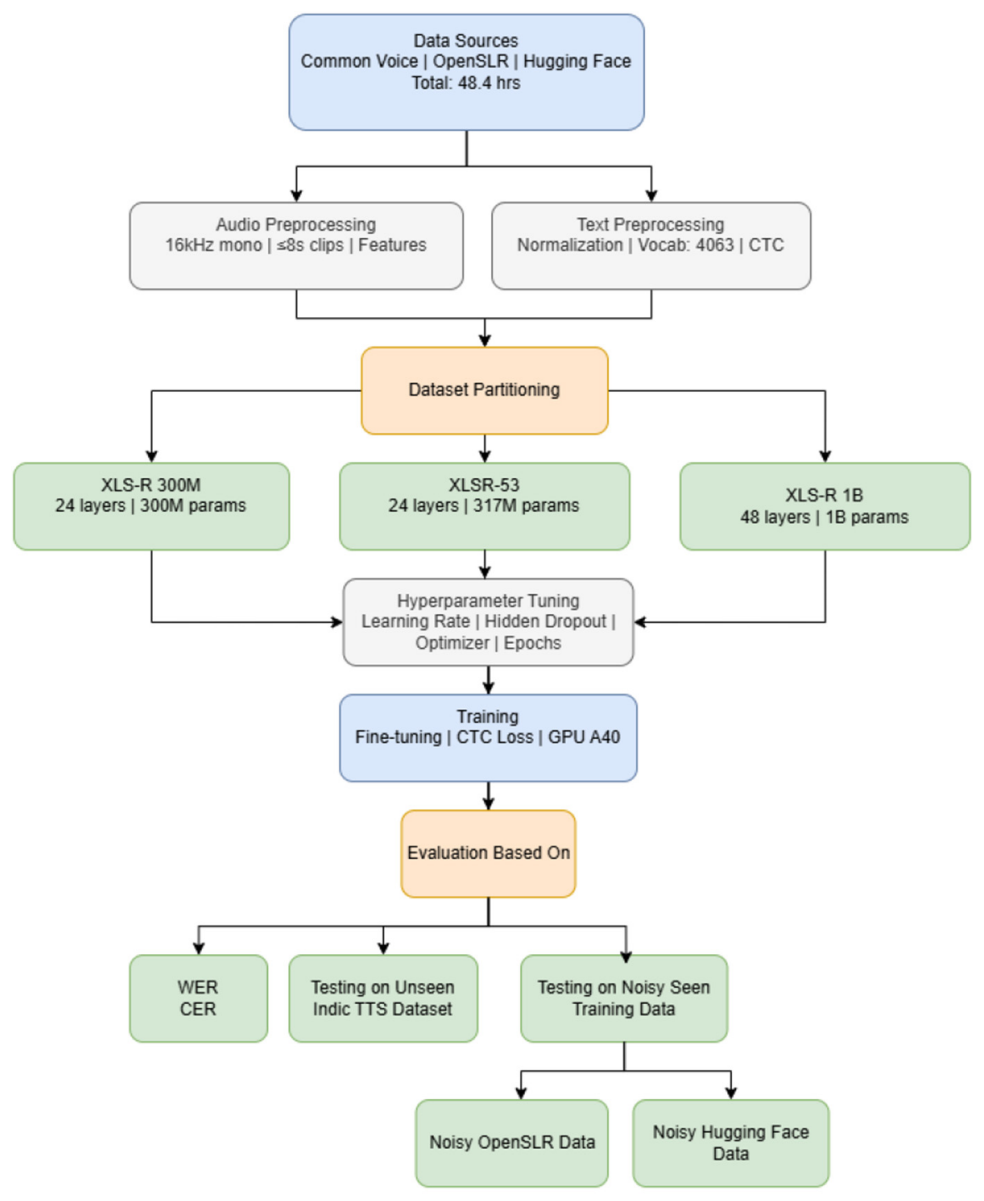
Overview of methodology framework.

This section provides a detailed overview of the methodology employed in this study, which includes dataset creation, data preprocessing, model tuning, and hyperparameter optimization. This multi-perspective method was critical in determining configurations that would give lower WER and CER with trade-offs between model size and computational tractability. Figure 1 depicts the methods used in this investigation.

### 3.1 Dataset description

The development of an ASR system in the Telugu language is challenging due to the lack of large and standardized datasets. To address this problem, a combined dataset was created to form a large dataset to train on. The datasets used in building a corpus were made up of three open-source repositories: Mozilla Common Voice (Ardila et al., 2020), OpenSLR (He et al., 2020), and the Hugging Face dataset (zsy12345/telugu-asr) (zsy12345, 2022) to increase phonetic and linguistic diversity. The total duration of audio in the combined dataset used to train totaled approximately 48.4 h. The combined dataset was partitioned using a 9:1 train-test split. During fine-tuning, a portion of the training data was internally used for validation to monitor model convergence and validation loss.

The datasets collected were from various sources, which included unique characteristics that contributed to the final combined dataset. The Hugging Face dataset had the largest amount of speech, which was over 40 h, and appeared to be formulated from a news broadcast on political and current events, therefore having a more professional speaking tone and specialized vocabulary. This was evident in the transcripts that contain political updates, public announcements, and current events. The Mozilla Common Voice dataset contributed 2.69 h of crowdsourced audio, which introduced a diversity of accents and acoustic environments. The OpenSLR dataset, which provided 5.71 h of audio, is a high-quality corpus of transcribed sentences recorded by volunteers. The Mozilla Common Voice dataset is the most informative with respect to demographics, providing metadata that indicates the audio recordings were 65% male speakers and 17% female speakers, while the remaining recordings did not contain gender metadata. It also includes demographic data for contributor ages, with the contributors between the ages of 20–29 comprising the majority. The OpenSLR corpus did not provide specific speaker percentages, but it did provide the data in separate archives for male speakers and female speakers, confirming it was a multi-gender corpus. The Hugging Face dataset did not publish any demographic features regarding the contributors, with gender being one of them. The following Table 1 summarizes the dataset statistics.

**Table 1:** Dataset statistics.

Attribute	Common voice	Hugging face	OpenSLR	Combined
Gender (F)	17%	–	51.6%	–
Gender (M)	65%	–	48.4%	–
Gender (Unspecified /Other)	18%	–	–	–
Sentences	2,311	20,698	4,448	27,457
Words	26,823	897,456	62,456	986,735
Unique words	483	3,759	1,205	4,063
Phonemes	35,061	1,153,593	80,403	1,269,057
Unique phonemes	42	58	56	67
Hours	2.69	40.00	5.71	48.40

### 3.2 Data preprocessing and unification

To maintain consistency, all audio clips were pre-processed in the same way. All audio data was reduced to a 16 kHz mono WAV file and converted to a uniform format. Secondly, the data was filtered to only include audio clips of 8 s or shorter. This threshold was chosen to maintain manageable audio lengths and reduce the usage of GPU VRAM during model training. Upon filtering, the combined dataset displayed a total of 20,576 files that had a total duration of 1,686.28 min. This dataset contained 4,063 unique words and 67 unique phonemes, providing adequate lexical and phonetic coverage to capture variations in speech during Telugu ASR training. The audio clips, along with their corresponding transcriptions, were then extracted and organized into a structured JSON format.

Telugu, as an Indic script, includes a wide range of diacritics, numerals, and special modifiers that are not always relevant in the context of phoneme-level speech recognition. Therefore, the text normalization process involved a deterministic string-substitution pass to remove non-essential elements while preserving core phonetic structures (Sunitha and Devi, 2013).

Specifically, the discarded character classes included: (i) Telugu digits (representing the localized baseline numerals zero through nine); (ii) archaic, obsolete, or rare script variants and vocalic modifiers, namely *Ardhachandrabindu* (nasalization sign), *Avagraha* (prolongation marker), historical fractional or non-standard vocalic signs, and specific archaic consonant variants; (iii) foreign Latin typographic script characters introduced accidentally as remnants during data ingestion; and (iv) standard alphanumeric punctuation marks, smart single and double quotes, arithmetic operators, dynamic string delimiters, and explicit structural control markers (such as zero-width non-joiners and trailing newline characters).

Conversely, all phonemically critical components were strictly preserved to maintain phonetic integrity. This includes primary independent vowels, core consonants, the *Anusvara*, all primary dependent vowel signs (*matras*), and the *Virama* or halant, which is essential for defining consonant clusters and preventing phonetic collapse during tokenization.

Post-normalization, the corpus integrity was validated by extracting the unique character vocabulary set across the combined datasets. This set was manually audited to guarantee that no phonetically vital graphemes were truncated and that the semantic/phonemic integrity of the text was preserved. A custom vocabulary was then built from this cleaned text corpus by mapping each unique character to a unique ID, resulting in a clean vocabulary of 67 unique tokens. Additional special tokens were also included:

- | for space
- [UNK] for unknown characters
- [PAD] for padding sequences

The cleaned text was then tokenized using a tokenizer compatible with Connectionist Temporal Classification (CTC) loss, and the audio inputs were transformed using the Wav2Vec 2.0 feature extractor, which normalized the raw waveforms and extracted relevant features. These features were then grouped into batches of consistent size using a custom data collator that dynamically handled padding during training (Wolf et al., 2020).

### 3.3 Wav2Vec 2.0 model architecture

The Wav2Vec 2.0 model architecture is based on the Transformer encoder, which allows for self-supervised learning, considered highly advantageous as most traditional STT models rely on labeled data, which can be difficult to gather in volume. The Wav2Vec 2.0 model leverages vast amounts of unlabeled audio data through self-supervised pre-training to learn robust speech representations. This approach is particularly effective for achieving high performance even when limited labeled data is available, demonstrating the feasibility of speech recognition in low-resource settings (Baevski et al., 2020). The architecture involves the four modules.

#### 3.3.1 Feature encoder

The feature encoder is a CNN that processes raw audio input into latent speech representations. It consists of seven blocks of temporal convolutions, each followed by layer normalization and a Gaussian Error Linear Unit (GELU) activation function. The output sequence *Z* = {*z*_1_, *z*_2_, …, *z*_*T*_} serves as input to the transformer network, preserving temporal relationships while significantly compressing the input space.

#### 3.3.2 Quantization module

Wav2Vec 2.0 learns discrete speech units, which are used to represent latent representations in its contrastive task. This is achieved through differentiable quantization utilizing Gumbel-Softmax reparameterization. The approach employs *G* codebooks with *V* entries each, where discrete selection is achieved via Equation 1:


$$\begin{array}{l} i_{g}=\arg\max\left(\sigma_{g,v}+\eta_{v}\right) \end{array}$$ (1)

with η_*v*_ = −log(−log(*u*_*v*_)) and *u*_*v*_ ~ *U*(0, 1).

The probability distribution is defined as Equation 2:


$$\begin{array}{l} p_{g,v}=\frac{\exp\left(\left(\sigma_{g,v}+\eta_{v}\right)/\tau \right)}{\sum_{k=1}^{V}\exp\left(\left(\sigma_{g,k}+\eta_{k}\right)/\tau \right)} \end{array}$$ (2)

where σ_*g, v*_ represents logits, τ is a non-negative temperature parameter, and η denotes Gumbel noise. During forward propagation, hard selection is applied, while backpropagation uses the continuous approximation. This straight-through estimator enables end-to-end differentiability. Selected entries $e_{g}^{i_{g}}$ are concatenated and linearly transformed to produce the final quantized vector *q* ∈ ℝ^*f*^, maintaining gradient flow for optimization.

#### 3.3.3 Content network

The transformer encoder processes latent feature vectors *Z* = {*z*_1_, *z*_2_, …, *z*_*T*_} from the convolutional feature encoder through multiple transformer blocks. Unlike standard transformers that require explicit positional embeddings due to the permutation-invariant nature of self-attention, Wav2Vec 2.0 employs convolutional positional embeddings (Ghosh et al., 2020; Podila et al., 2025). This architecture learns relative positional encodings directly from the audio sequence, capturing local dependencies while generating position-aware contextual representations *C* = {*c*_1_, *c*_2_, …, *c*_*T*_}. By integrating these learned embeddings into the transformer input, the model preserves temporal ordering essential for speech recognition without relying on fixed sinusoidal embeddings, effectively capturing relative positional relationships throughout the processing hierarchy.

#### 3.3.4 Pretraining objective

The self-supervised pretraining aims to learn robust speech representations by masking latent feature vectors and reconstructing them through contrastive prediction. The training employs two complementary loss functions:

- Contrastive loss: the model identifies the correct quantized vector q_*t*_ (corresponding to the masked timestep *t*) among *K*+1 distractors by maximizing cosine similarity with context representation **c**_*t*_ (Equation 3):
$$\begin{array}{l} L_{\text{contrast}}=-\log\frac{\exp\left(\text{sim}\left({\text{c}}_{t},{\text{q}}_{t}\right)/\tau \right)}{\sum_{\tilde{\text{q}}\sim Q_{t}}\exp\left(\text{sim}\left({\text{c}}_{t},\tilde{\text{q}}\right)/\tau \right)} \end{array}$$ (3)
  where τ denotes the temperature parameter and *Q*_*t*_ contains *K* negative samples plus the positive sample q_*t*_.
- Diversity loss: to prevent codebook collapse and encourage uniform codebook utilization, a diversity loss maximizes entropy (Equation 4):
$$\begin{array}{l} L_{\text{div}}=\frac{1}{GV}\overset{G}{\underset{g=1}{\sum }}\overset{V}{\underset{v=1}{\sum }}{\bar{p}}_{g,v}\log{\bar{p}}_{g,v} \end{array}$$ (4)
  where *V* represents entries per codebook, *G* is the number of codebooks, and ${\bar{p}}_{g,v}$ is the mean softmax probability over batches.

The composite pretraining loss combines both objectives (Equation 5):


$$\begin{array}{l} L=L_{\text{contrast}}+\lambda L_{\text{div}} \end{array}$$ (5)

where λ is a tunable hyperparameter controlling regularization strength.

### 3.4 Training protocol and hyperparameters

To ensure reproducibility, model execution followed a deterministic training protocol. All model variants were optimized using the AdamW optimizer across a total of 15 epochs. The training parameters were configured with a per-device batch size of 8 and a gradient accumulation strategy of two steps, establishing an effective global batch size of 16 samples. The initial peak learning rate was set to 3 × 10^−4^, utilizing a linear learning rate decay schedule preceded by a linear warm-up phase covering the first 10% of the total training steps (warmup_ratio = 0.1).

Due to the continuous token-length nature of speech frames and the primary sequence objectives, dataset partitioning for the train and validation sets was implemented using a randomized split (90/10 distribution) stabilized by a fixed random seed initialization (seed = 42) rather than categorical stratification. Models completed their training routine fully across the predefined epoch ceiling to maximize parameter optimization convergence.

### 3.5 Hyperparameter study

In this study, we analyze three variants of the Wav2Vec 2.0 model–XLS-R-300M, XLSR-53, and XLS-R-1B–through an ablation study to examine how changes in key hyperparameters affect model performance. Below is a brief overview of each hyperparameter chosen for this study.

#### 3.5.1 Learning rate

The learning rate controls the step size used when updating model weights during training. Choosing an appropriate value is critical, as it directly affects the efficiency and stability of model training. A high learning rate can lead to unstable training or divergence, whereas a low learning rate can cause very slow convergence and increase the risk of the model becoming stuck in a local minimum (Ruder, 2016). In this study, learning rates of 3 × 10^−4^, 5 × 10^−5^, and 1 × 10^−4^ were employed to determine the optimal configuration for fine-tuning.

#### 3.5.2 Hidden dropout

Dropout is a regularization technique used to prevent overfitting. This hyperparameter manages the dropout rate for each layer in the neural network. A lower dropout rate allows for more detailed learning but increases the risk of overfitting. Conversely, a higher dropout rate can lead to underfitting, where the model fails to capture relevant patterns in the data (Srivastava et al., 2014). Tuning the hidden dropout ratio helps achieve a balance between memorization and generalization that is well-suited to the characteristics of the dataset. For the hidden dropout rate, the models were configured with dropout rates of 0.1, 0.2, and 0.3, respectively, to identify the optimal setting.

#### 3.5.3 Optimizer

Optimizers determine how the model's weights are updated during training based on gradient calculations. Modern natural language processing models often use adaptive optimizers, which automatically adjust learning rates for different parameters (Ruder, 2016). The optimizers explored in this study include:

- Adam and its variant Adam W.
- Adafactor: a memory-efficient alternative well-suited for large-scale models (Shazeer and Stern, 2018).

By comparing these optimizers, we aim to identify which ones lead to faster convergence and better final accuracy across the different model variants.

#### 3.5.4 Epochs

One epoch refers to a single, complete pass through the entire training dataset. Increasing the number of epochs allows the model to refine its learned patterns but also increases the risk of overfitting if the value is too high. Too few epochs can result in an undertrained model that fails to capture important data patterns (Hussein and Shareef, 2024). In this work, we experimented with 15, 25, and 30 epochs to determine the optimal number of training epochs for the three variants of Wav2Vec 2.0.

### 3.6 Evaluation metrics

To evaluate the performance of all models, we used two standard metrics for evaluating the performance of ASR: WER and CER, which measure the distance between predicted text and original reference, and a lower result indicates higher accuracy.

#### 3.6.1 Word error rate (WER)

The WER calculates errors at the word level. The formula is based on the Levenshtein distance, which counts the minimum number of edits required to change the prediction of the model into the reference transcript. The formula for WER is:


$$\text{WER}=\frac{S+D+I}{N}$$

where:

- S is the number of substitutions, where a word in the reference is incorrectly replaced with another word.
- D is the number of deletions, where a word in the reference is missing from the hypothesis.
- I is the number of insertions, where a word is present in the hypothesis but not in the reference.
- N is the total number of words in the reference transcript.

#### 3.6.2 Character error rate (CER)

The CER evaluates errors at the character level. It is useful for calculating performance in languages that have more complexities and unclear word boundaries. The formula for CER is:


$$\text{CER}=\frac{S_{c}+D_{c}+I_{c}}{N_{c}}$$

where:

- *S*_*c*_ is the number of character substitutions.
- *D*_*c*_ is the number of character deletions.
- *I*_*c*_ is the number of character insertions.
- *N*_*c*_ is the total number of characters in the reference transcript.

### 3.7 Computational costs

The training of the base model and all ablation experiments were done using a cloud based instance on Runpod. We deployed an A40 pod, which featured a single NVIDIA A40 graphics processing unit (GPU) with 48 GB of VRAM, paired with nine virtual central processing unit cores and 48 GB of system RAM. It had 40 GB of disk storage, and the software was set up in a Docker container, where a Jupyter notebook was used for running PyTorch version 2.1.0 (PyTorch Foundation, USA.) on an Ubuntu 22.04 operating system with CUDA 11.8.0. The cost of this pod template per use was $0.40 per hour for the GPU and $0.006 per hour for storage. The approximate training duration for the XLS-R-300M and XLS-R-53 models was approximately 6 h each, while the XLS-R-1B model required around 10 h under the experimental setup.

## 4 Results and discussions

This section presents the results and findings on the effect of different hyperparameters on the performance of the XLS-R-300M, XLSR-53, and XLS-R-1B models. The hyperparameters considered include learning rate, hidden dropout, optimizer, and number of epochs. Each finding is discussed in detail, and the performance of the models is compared on the testing dataset to assess the final performance. While data preparation steps such as dataset partitioning were controlled using fixed random initialization seeds, all downstream Word Error Rate (WER) and Character Error Rate (CER) metrics reported are single-point estimates from single execution runs due to cloud-based computational resource constraints. Consequently, any marginal differences in performance metrics between model variants should be interpreted with care when claiming model superiority.

### 4.1 Effect of learning rate

To find the optimal value, the learning rate was assessed at three distinct levels for the models, with the quantitative findings detailed in Table 2. For the XLS-R-300M model, the best performance was considered with a learning rate of 5 × 10^−5^, resulting in the WER of 0.3825 and the lowest CER of 0.1605. Although the WER achieved with a learning rate of 1 × 10^−4^ was lower, 5 × 10^−5^ was chosen because it achieved the best CER, and the very small difference in WER (only 0.0013) was considered negligible. Similarly, the XLSR-53 model exhibited its best performance at a learning rate of 5 × 10^−5^, achieving a WER of 0.3790 and CER of 0.1584. In the case of the XLS-R-1B model, the best learning rate was determined to be 1 × 10^−4^, which led to the lowest WER of 0.3653 and CER of 0.1557.

**Table 2:** Effect of the learning rate on the performance of WER and CER for the XLS-R-300M, XLSR-53, and XLS-R-1B models.

Learning rate	XLS-R-300M		XLSR-53		XLS-R-1B	
	WER	CER	WER	CER	WER	CER
3 × 10^−4^ (base_model)	0.4099	0.1664	0.3859	0.1600	0.4050	0.1648
5 × 10^−5^	0.3825	0.1605	0.3790	0.1584	0.3685	0.1573
1 × 10^−4^	0.3812	0.1619	0.3804	0.1606	0.3653	0.1557

**Figure 2:**
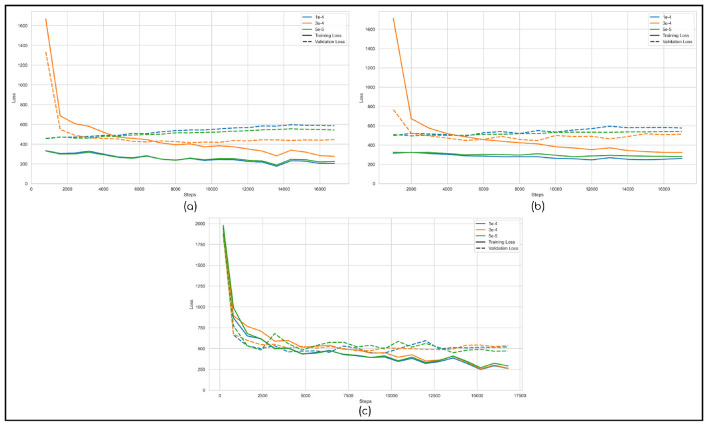
Loss curves for different learning rates across models. **(a)** XLS-R-300M. **(b)** XLSR-53. **(c)** XLS-R-1B.

The loss curves of different learning rates of the models are shown in Figure 2. For the XLS-R-300M model, the loss curves suggest that the learning rate of 5 × 10^−5^ was necessary for its superior performance. The slower and more stable convergence at this rate prevented the model from settling into a suboptimal minimum, thereby facilitating lower error rates and improved generalization, despite other rates converging at a faster pace. The loss curves for the XLSR-53 model indicate that a learning rate of 5 × 10^−5^ struck an optimal balance between stability and convergence speed, resulting in a more generalized model and reduced WER. For the XLS-R-1B model, the loss curves across all tested learning rates were marked by noise. Nevertheless, the learning rate 1 × 10^−4^ consistently exhibited the lowest average validation loss during the later stages of training. This improvement in training performance corresponds with its attainment of the best final WER and CER.

### 4.2 Effect of hidden dropout rate

To find the optimal value, the hidden dropout rate was assessed at three different levels for the models, with the quantitative findings detailed in Table 3. For the XLS-R-300M model, the lowest WER of 0.3787 and CER of 0.1581 were attained with a hidden dropout of 0.3. The XLSR-53 achieved the best results for a hidden dropout of 0.2 with a WER of 0.3778 and a CER of 0.1581. For the XLS-R-1B model, a hidden dropout rate of 0.1 gave the lowest WER of 0.3653 and CER of 0.1557.

**Table 3:** Effect of the hidden dropout rate on the performance of WER and CER for the XLS-R-300M, XLSR-53, and XLS-R-1B models.

Hidden dropout rate	XLS-R-300M		XLSR-53		XLS-R-1B	
	WER	CER	WER	CER	WER	CER
0.1	0.3825	0.1605	0.3790	0.1584	0.3653	0.1557
0.2	0.3797	0.1584	0.3778	0.1581	0.3778	0.1572
0.3	0.3787	0.1581	0.3850	0.1604	0.3892	0.1615

**Figure 3:**
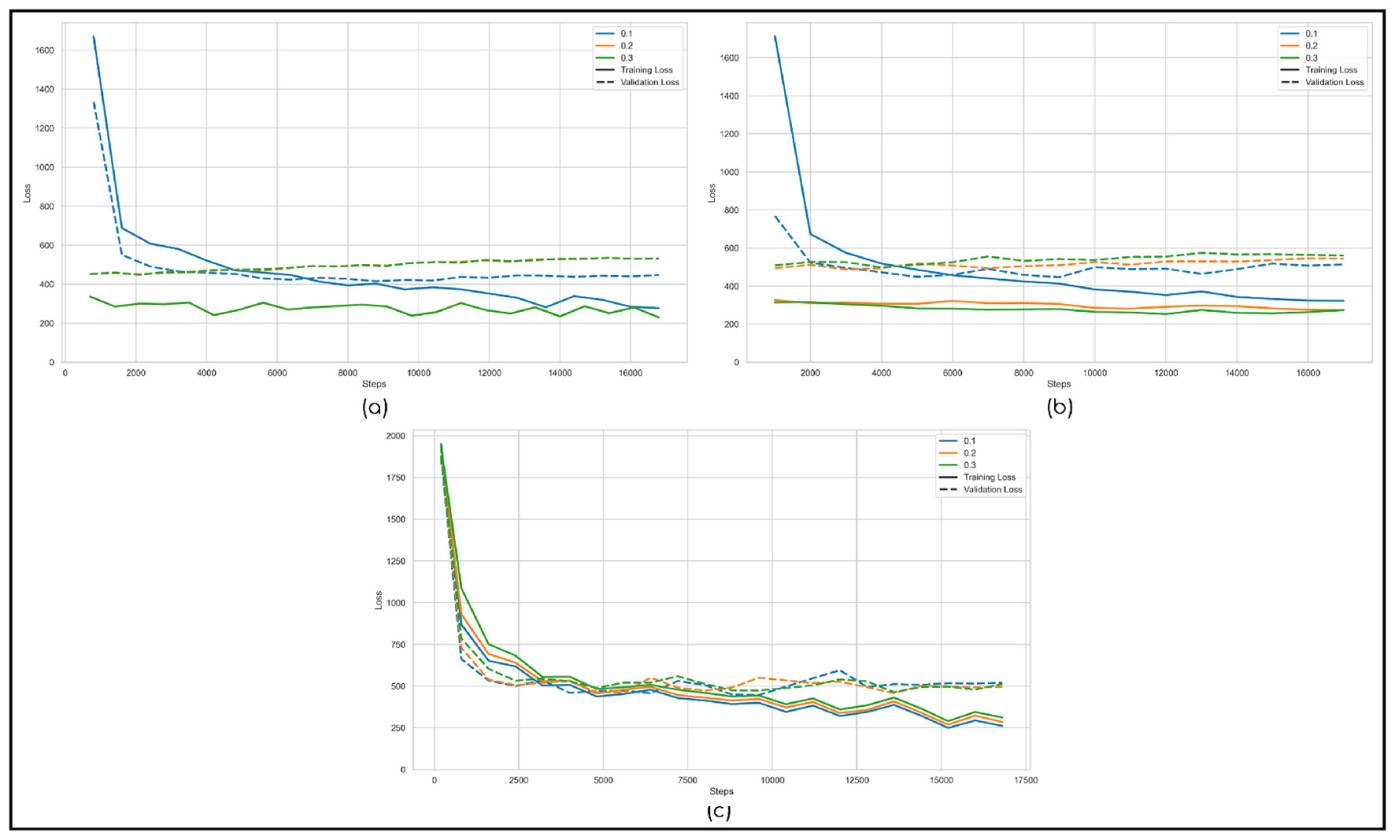
Loss curves for different hidden dropout rates across models. **(a)** XLS-R-300M. **(b)** XLSR-53. **(c)** XLS-R-1B.

The loss curves of the models are shown in Figure 3. Analyzing the loss curves for the XLS-R-300M indicates that a dropout rate of 0.1 exhibits signs of overfitting, as its validation loss stabilizes much above its consistently decreasing training loss. In contrast, increasing the dropout to 0.3 offered robust regularization. Despite a higher in-training loss, this regularization was the most effective, ultimately resulting in the best generalization. For the XLSR-53 model, the loss curves imply that a dropout rate of 0.1 was marginally under-regularized, while 0.3 was excessively aggressive. The rate of 0.2 provided the optimal regularization, providing the best quantitative outcome. Regarding the XLS-R-1B model, the 0.1 dropout model demonstrates the best generalization. On the other hand, both the training and validation lines for the 0.2 and 0.3 dropout models are visibly higher, which indicates signs of over-regularization.

### 4.3 Effect of optimizers

**Figure 4:**
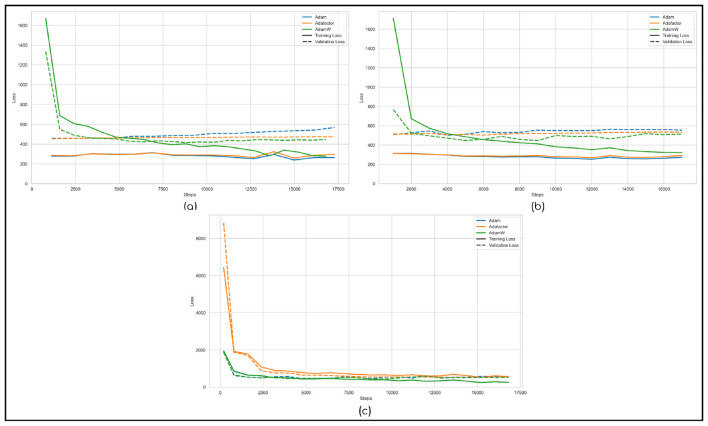
Loss curves for different optimizers across models. **(a)** XLS-R-300M. **(b)** XLSR-53. **(c)** XLS-R-1B.

To determine the optimal value, the optimizers were evaluated at three levels for the models, with the quantitative results presented in Table 4. The XLS-R-300M model demonstrated optimal performance when utilizing the AdamW optimizer, resulting in a WER of 0.3787 and a CER of 0.1581. For the XLSR-53 model, AdamW also yielded the best results, with a WER of 0.3778 and a CER of 0.1581. For the XLS-R-1B model, the best-performing optimizer was Adam, which achieved a WER of 0.3623 and a CER of 0.1544. The loss curves of the models are shown in Figure 4. A detailed analysis of the training process for the XLS-R 300M model indicates that both the AdamW optimizer and the Adam optimizer exhibit significant initial convergence. Throughout the training duration, AdamW consistently achieves a lower and more stable validation loss in comparison to Adam. In contrast, the Adafactor optimizer presents a notably flat loss profile. The effectiveness of the AdamW optimizer is confirmed by the final metrics shown in the table, with WER 0.3787.

**Table 4:** Effect of optimizers on the performance of WER and CER for the XLS-R-300M, XLSR-53, and XLS-R-1B models.

Optimizer	XLS-R-300M		XLSR-53		XLS-R-1B	
	WER	CER	WER	CER	WER	CER
Adam	0.3807	0.1661	0.3844	0.1601	0.3623	0.1544
AdamW	0.3787	0.1581	0.3778	0.1581	0.3653	0.1557
Adafactor	0.3854	0.1594	0.3864	0.1595	0.5597	0.2038

The best results for the XLSR-53 model were seen using the AdamW optimizer. TThe loss curves further support this observation, with AdamW maintaining more consistent convergence behavior than the conventional Adam optimizer. The preference for AdamW was further supported by the fact that, although maintaining a consistent profile, the Adafactor optimizer did not match the optimal final error rate. In the case of the XLS-R 1B model, the loss curves and final metrics are in evident agreement. Both Adam and AdamW exhibited comparable, robust performance; however, the marginally superior convergence behavior and lower final validation loss of Adam resulted in the most favorable results.

The poor performance of Adafactor, particularly for XLS-R-1B, may be associated with optimization instability during low-resource fine-tuning of large transformer models. Although Adafactor is designed as a memory-efficient optimizer (Shazeer and Stern, 2018), prior studies have noted that adaptive optimizers can show less stable convergence behavior in limited-data fine-tuning settings compared to Adam-based approaches Ruder (2016). In this work, Adafactor-specific parameters, including relative step sizing and clipping, were retained at their default settings to ensure consistent optimizer comparison across experiments. The substantially higher validation loss and final error rates observed for XLS-R-1B using Adafactor compared to AdamW indicate that Adafactor was less suitable for this low-resource Telugu ASR setting.

### 4.4 Effect of epochs

To determine the optimal value, the number of epochs was evaluated at three levels for the models, with the quantitative results presented in Table 5. For the XLS-R-300M model, performance improved with longer training, reaching the best result at 30 epochs with a WER of 0.3714 and CER of 0.1608. For the XLSR-53 model, performance similarly improved with increased training time, achieving the lowest WER of 0.3768 and CER of 0.1608 at 30 epochs. We considered 15 epochs as the optimal duration for these two models because extended training provided negligible improvements to WER while simultaneously causing the CER to increase. Similarly, for the XLS-R-1B model, the best performance was observed at 15 epochs with a WER of 0.3623 and CER of 0.1544.

**Table 5:** Effect of epochs on the performance of WER and CER for the XLS-R-300M, XLSR-53, and XLS-R-1B models.

Number of training epochs	XLS-R-300M		XLSR-53		XLS-R-1B	
	WER	CER	WER	CER	WER	CER
15	0.3787	0.1581	0.3778	0.1581	0.3623	0.1544
25	0.3783	0.1601	0.3799	0.1599	0.3630	0.1586
30	0.3714	0.1608	0.3768	0.1608	0.3635	0.1627

**Figure 5:**
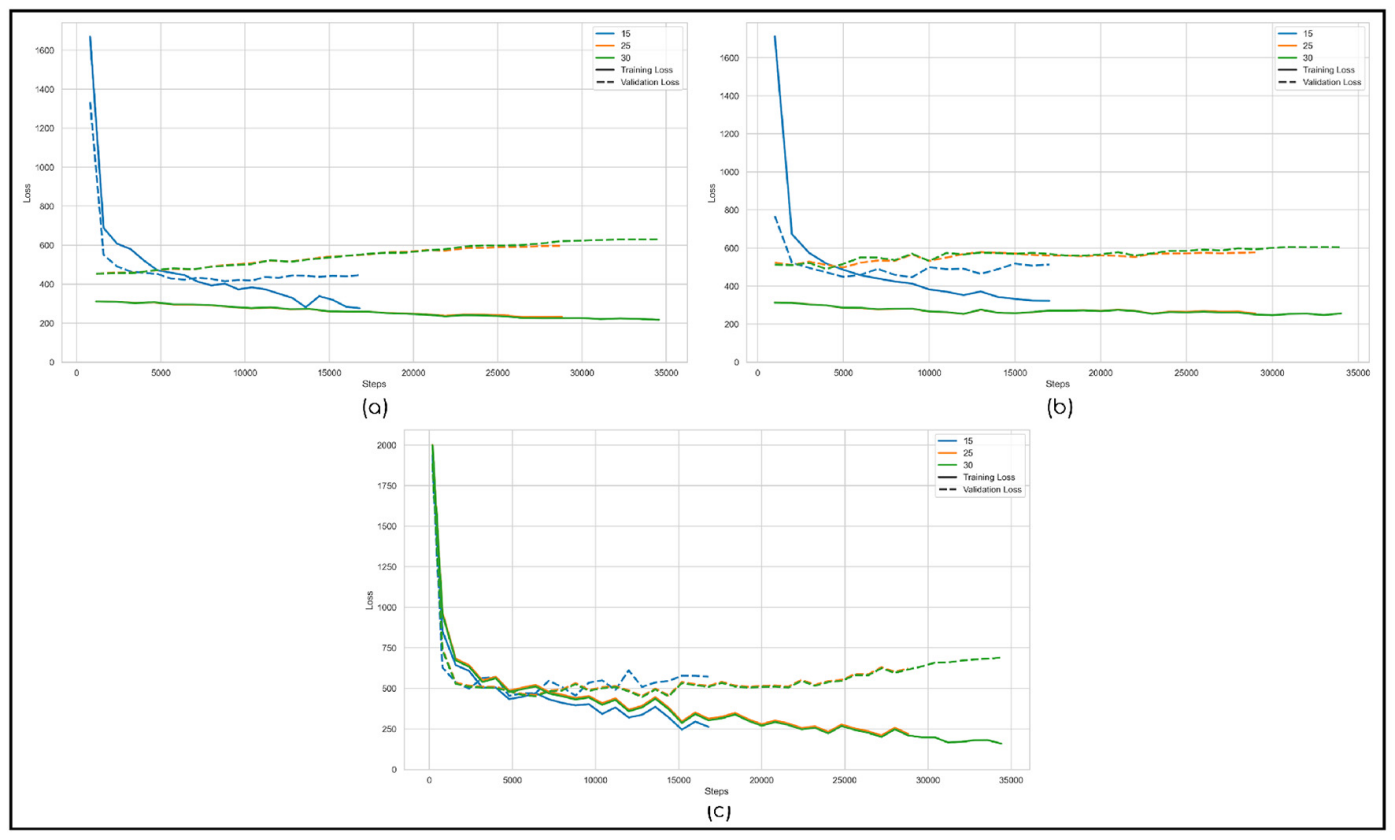
Loss curves for different numbers of epochs across models. **(a)** XLS-R-300M. **(b)** XLSR-53. **(c)** XLS-R-1B.

The loss curves of the models are shown in Figure 5. Both the XLS-R-300M and the XLSR-53 models indicate that following the 15-epoch mark, the gap begins to widen between the two loss lines in the graph. The solid training line continues to fall gradually, while the dashed validation line begins to stall and trend upwards slowly. Hence, the widening gap is a classic visual indicator of the beginning of overfitting. This effect is more pronounced and immediate with the XLS-R-1B model. This graph indicates that the validation loss has clearly taken a “V” shape, reaching its minimum value of 520.7419 at approximately epoch 12.91 (step 15,000). Immediately following this instance, the dashed validation line is on a sharp and clear incline, indicating that continued training no longer offers additional benefit and instead negatively affects the model's performance through overfitting.

### 4.5 Cross-dataset generalization and robustness

To evaluate the real-world applicability of our fine-tuned Wav2Vec 2.0 variants, we conducted a cross-dataset generalization analysis. Evaluating models against unseen data distributions is critical for deployment, as distribution shifts in acquisition and acoustic environments will be present. In this study, our models were trained on a curated 48.4-h multisource corpus–integrating data from Mozilla Common Voice, OpenSLR, and Hugging Face. By combining data from different sources, we prevent the model from just memorizing the dataset, or over-fitting.

Furthermore, to explicitly test against out-of-domain distribution shifts, we utilized the unseen Indic TTS dataset as an evaluation benchmark.We validated the robustness of our models by testing them against the distinct acoustic profiles of the Indic TTS dataset. Maintaining strong performance across such distribution shifts demonstrates their true generalizability and practical value for Telugu ASR.

### 4.6 Model performance summary

After conducting the ablation studies to identify the optimal hyperparameters for each model variant, we trained each model with its best configuration. The final performance on the testing data is summarized in Table 6. The XLS-R-300M model achieved a WER of 0.3787 and a CER of 0.1581. The XLSR-53 model yielded a WER of 0.3778 and a CER of 0.1581, while the XLS-R-1B model achieved a WER of 0.3623 and a CER of 0.1544.

**Table 6:** Performance summary of models on the test set.

Model	WER	CER
XLS-R-300M	0.3787	0.1581
XLSR-53	0.3778	0.1581
XLS-R-1B	0.3623	0.1544

## 5 Qualitative study

This section presents the performance of the fine-tuned model on unseen and noisy data, along with a detailed error analysis.

### 5.1 Performance on unseen data

To begin, the models were evaluated on 6.70 h of the completely unseen Indic TTS dataset (Speech Technology Consortium et al., 2023) to assess the models' robustness. The Indic TTS dataset was not included during training. The fine-tuned XLS-R-300M, XLSR-53, and XLS-R-1B models demonstrated satisfactory performance on this dataset, as presented in Table 7.

**Table 7:** Testing results on unseen Indic TTS dataset.

MODEL	WER	CER
XLS-R-1B	0.3471	0.0717
XLS-R-300M	0.3397	0.0662
XLSR-53	0.3264	0.0609

### 5.2 Performance on seen data with noise augmentation

The fine-tuned models were evaluated on the OpenSLR and Hugging Face training datasets with added noise to assess model robustness. Background noise was sourced from the ESC-50 dataset (Piczak, 2015). To isolate the acoustic origin as the primary variable, identical noise types were applied across both evaluations.

The first evaluation used the OpenSLR dataset, with results presented in Table 8, yielding a WER of 19.74% for the XLS-R-300M model. The second evaluation was conducted on the Hugging Face dataset, with results shown in Table 9, where performance degraded sharply to a WER of approximately 48.9%.

**Table 8:** Testing results on OpenSLR data with noise augmentation.

MODEL	WER	CER
XLS-R-1B	0.5169	0.2221
XLS-R-300M	0.1974	0.0578
XLSR-53	0.2299	0.0719

**Table 9:** Testing results on the Hugging Face dataset with noise augmentation.

MODEL	WER	CER
XLS-R-1B	0.4663	0.2012
XLS-R-300M	0.4893	0.2214
XLSR-53	0.4769	0.2163

This 27-point performance gap is attributed to the intrinsic acoustic differences between the two corpora. While OpenSLR consists of clean, standardized, high-quality read speech, the Hugging Face corpus contains crowd-sourced recordings featuring diverse microphone profiles, variable acoustic environments, and aggressive audio compression artifacts. When environmental noise is injected into the already degraded, low-fidelity signals of the Hugging Face dataset, a compounding distortion occurs that severely disrupts phoneme-grapheme alignment. This divergence highlights critical implications for real-world deployment: robustness metrics derived purely from controlled, studio-quality speech data fail to generalize to unconstrained, heterogeneous noise environments where intrinsic channel degradation is present.

### 5.3 Error analysis on unseen data

**Figure 6:**
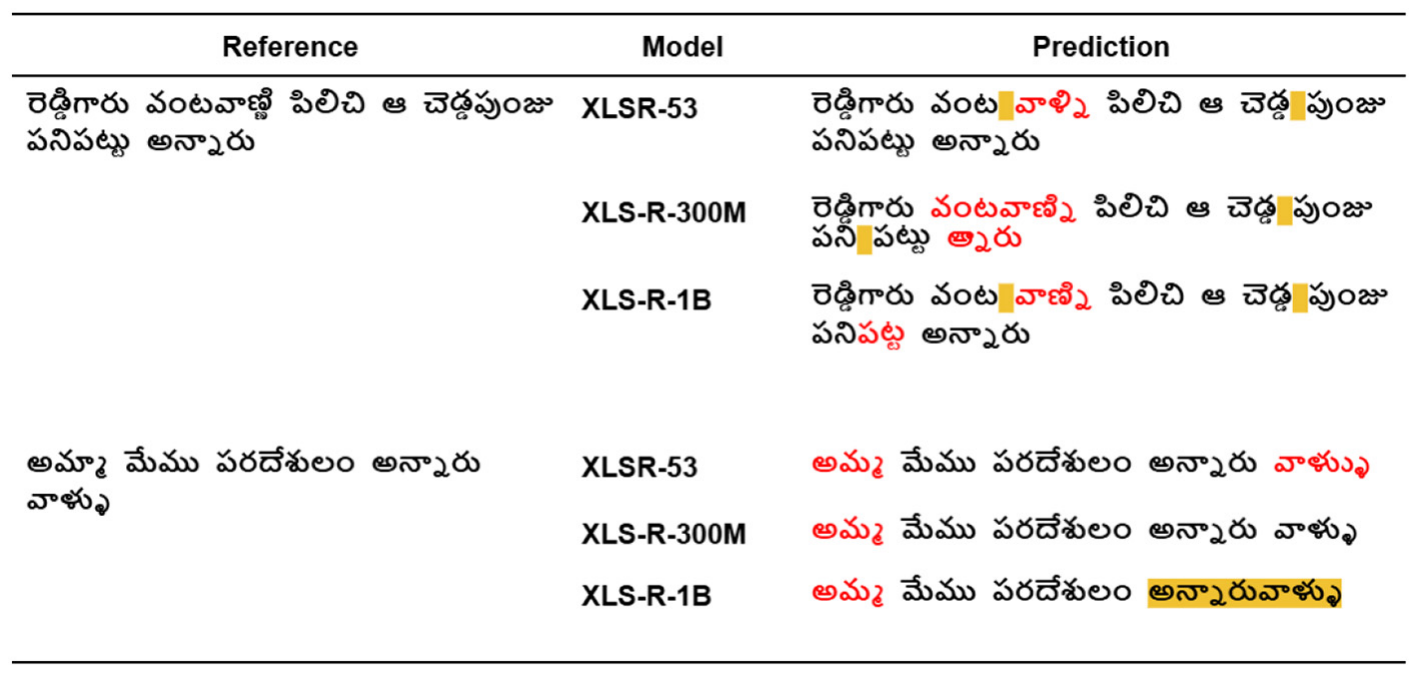
Comparative transcription errors for all three models.

To better understand the models' performance on the unseen Indic TTS dataset, we analyzed their specific transcription errors. The following analysis, with examples shown in Figure 6, highlights the spacing errors in yellow and word error patterns in red for each model.

XLSR-53 made a compound error on the Telugu word for “cook” (vaṇṭavāṇṇi). It interchanged the nasal ‘ṇ‘a' sound with the ‘ḍ‘a' sound. Additionally, the model failed on the word ammā (“mother”) by not capturing the length of the final vowel, transcribing the long ‘ā‘' sound as a short ‘a' (amma). XLS-R-300M performed slightly better on the word for “cook” by correctly keeping it as a single word, but it still made the same phonetic substitution of the ‘ṇ‘a' for the ‘ḍ‘a' sound. It also consistently failed on the word ammā, making the same error as the other models by shortening the final long vowel. XLS-R-1B exhibited errors that were identical to those of the XLSR-53 model. It also made an error in making the consonant substitution. Similarly, it failed to transcribe the final long vowel in the word ammā.

Across the analyzed IndicTTS samples and predictions, substitution errors were the most frequently observed error type for all three models, while insertion errors were comparatively rare. Other errors included vowel length reductions, consonant substitution, and improper handling of morphologically rich Telugu words. The models also showed inconsistencies in compound word segmentation and spacing, where words that should have been kept as one were often split apart or combined with each other. The deletion errors were primarily due to the lack of or missing suffixes or partially truncated. In contrast, insertion errors were infrequent, usually as duplicated characters or additional tokens. The analysis suggests that phonetically similar sounds and complex compound word structure of Telugu remain challenging on unseen audio samples.

### 5.4 Analysis of results

The performance differences among the models can be attributed to their size and pre-training data:

- XLS-R-1B: this model performs well with clean, in-domain data, arguably as a result of its enormous parameter size that boosts its memorization ability. That same fact, however, makes it less resilient to the noisy conditions it was not specifically trained upon.
- XLS-R-300M: this model offered the optimal balance for noisy audio. It seems to generalize more by learning more robust and fundamental patterns and therefore is less prone to performance loss due to random noise.
- XLSR-53: this model performed best with the unseen Indic data because of its wide multilingual pre-training. This wide exposure enables it to learn to adapt better to the subtleties of a new language and is the most capable option for out-of-domain transcription tasks.

## 6 Conclusion

This research provided a comparative study of three Wav2Vec 2.0 variations, XLS-R-300M, XLSR-53, and XLS-R-1B, and their fine-tunings on a 48.4-h Telugu speech dataset. This reveals a visible difference in performance between the tested conditions, highlighting that the size and pretraining diversity of the model possess varying effects on robustness. The XLS-R-1B model significantly outperformed others on clean in-domain sets and thus confirmed the value of scale in modeling complex speech representations. However, the relatively unsatisfactory performance on noisy and unseen testing conditions highlights the natural drawback of overly parameter-rich models, susceptible as they are to overfitting and domain-mismatch difficulties. In contrast, the XLS-R-300M model best withstood noisy speech conditions, the XLSR-53 model robustly and invariably achieved better generalizability to unseen data–a result corroborative of the argument that smaller and better-balanced multilingual models are capable of better robustness in the face of distributional shift. These results agree with and do not go against expectations: there is no one “best" model regardless of conditions. Rather, the best model selection is conditional on deployment. The XLS-R-1B is best for clean, controlled applications; the 300M is optimal for noisy, realistic settings; and the XLSR-53 is the best for unseen domain generalization.

In sum, we present empirical proof that scaling alone is not enough for low-resource languages like Telugu; robustness is an intersection between model size, pretraining diversity, and evaluation context. We encourage future research to leverage these findings by investigating adaptive fine-tuning schedules, model ensembling, and noise-robust training methods to provide ASR systems with constant, accurate performance regardless of usage scenario. The full implementation code of this work can be accessed at: https://github.com/nikhitajames/Telugu_Wav2vec2.0_finetuning_and_ablation.

## Data Availability

Publicly available datasets were analyzed in this study. This data can be found here: https://commonvoice.mozilla.org/en/datasets; https://www.openslr.org/66/; https://huggingface.co/datasets/zsy12345/telugu-asr.
